# Institutional Responsibility is Prior to Personal Responsibility in a Pandemic

**DOI:** 10.1007/s10790-021-09876-0

**Published:** 2022-01-03

**Authors:** Ben Davies, Julian Savulescu

**Affiliations:** Oxford Uehiro Centre for Practical Ethics, Suite 8, Littlegate House, St Ebbe’s Street, Oxford, OX1 1PT UK

## Introduction

On 26 January 2021, while announcing that the country had reached the mark of 100,000 deaths within 28 days of COVID-19, UK Prime Minister Boris Johnson said that he took “full responsibility for everything that the Government has done” as part of British efforts to tackle the pandemic. The force of this statement was undermined, however, by what followed:

What I can tell you is that we truly did everything we could, and continue to do everything that we can, to minimise loss of life and to minimise suffering... [1]

Taking these sentiments together, it was hard to avoid the conclusion that the government took responsibility only for doing everything right. Such an admission of responsibility, accommodating no admission of error or wrongdoing, is not really about responsibility at all. Indeed, the UK’s pandemic response has been marked from its earliest days by a shifting of focus from *institutional* responsibility to personal responsibility for individual UK residents [2].

This paper explores the relationship between these two sites of responsibility—the institutional and the personal—in the context of the sort of pandemic that is ongoing as we write. Section 2 offers some initial definitions and discussions of key terms. Section 3 outlines how these two ideas interact in principle during such emergencies. Section 4 turns to the ‘non-ideal’ implications for personal responsibility of failures of institutional responsibility. While our focus is on the specific context with which we are most familiar, the UK governmental approach, where appropriate we also include examples from other states.

## Personal and Institutional Responsibility

Much discussion of responsibility in healthcare focuses on its potential role as a distributive criterion, e.g., whether it is right to treat patients differently when their health needs are due to their own choices. In a pandemic, certain ways of understanding this idea seem clearly ruled out. For instance, some might think that those who knowingly ignore pandemic guidelines, and who become ill, should not have access to treatment. Yet in a pandemic, a policy of refusing to treat people who are responsible for their condition would need to reckon with what happens to those infected with the SARS-CoV-2 virus who are not admitted to hospital. If they must be cared for by loved ones, this risks infection of *non*-culpable individuals. Thus, even ignoring powerful moral arguments against such a policy [3, 4], there are public protection grounds to reject it during a pandemic.

Even policies which are less extreme, such as prioritising access to critical care beds or ventilators, face pragmatic challenges. For instance, Davies and Savulescu [5] suggest that although there are grounds for ignoring responsibility when responsibility-sensitivity will make a patient very badly off, when we face a *choice* between patients who will both become badly off if not treated, and when resources are scarce, it may be legitimate to consider responsibility again. Yet the argument explicitly excludes emergencies, primarily because attempting to judge responsibility in an emergency would inevitably fall to medical staff, often resulting in worse outcomes for both culpable and non-culpable patients. Another important consideration raised by the current COVID pandemic is that medical staff are emotionally, physically and mentally overwhelmed. It would be immoral to ask them to make difficult judgements of responsibility on top of their already onerous workload. Thus, our discussion of responsibility has a different focus.

We are all affected by decisions of international bodies such as the World Health Organization and United Nations, and by the decisions of other countries. A pandemic is an international issue which requires global cooperation. Yet as individuals we are also affected by the decisions of our own national and regional institutions, and it is on these latter levels that we focus. Pandemics give rise to collective responsibilities which can be conceptually divided up in various ways. One important division is between the ‘personal responsibility’ of individuals, and the ‘institutional responsibility’ of bodies such as national and regional government, advisory bodies such as the UK’s Scientific Advisory Group for Emergencies (SAGE) and social bodies such as the National Health Service (NHS) and police forces. Our primary focus is on government and its advisors, but we also discuss other institutions where relevant. We do not suggest that institutional responsibility for an issue fully precludes personal responsibility. In a democracy, some elements of institutional responsibility are, at least indirectly, in our collective hands [6, 7], and each of us may have responsibilities to vote and advocate in ways that support better institutional responses. But at the more immediate stage, when a problem such as COVID confronts us, this kind of control that citizens have over their governments is far more limited.

Our interest is in how these two levels of responsibility interact. As already noted, UK government rhetoric has largely focused—the Prime Minister’s 26 January claim notwithstanding—on *personal* responsibility. For instance, in the brief period before reversing planned relaxations of lockdown rules over Christmas 2020, then-Health Secretary Matt Hancock made a speech in Parliament insisting that the public should take “personal responsibility” [8] for behaving in ways that the government had implicitly endorsed by relaxing restrictions but would soon go on to prohibit again [9]. In May 2020, the Prime Minister’s focus was not on governmental responsibility, but on “good, solid British common sense” [10]. More recently, Hancock blamed vaccine hesitancy for upsurges in cases of the Delta variant in various areas of the country [11] but did not acknowledge the possible role of a decision not to immediately put India on the UK’s ‘red list’ for quarantine rules [12].

One important distinction is between retrospective and prospective claims of responsibility. Prospective claims focus on, as Cane [13, p. 281] puts it, questions such as “what are our responsibilities?”. For instance, the claim that ‘we must all work together to beat this virus’ is a prospective claim of *collective national* responsibility. Retrospective responsibility concerns the apportionment of responsibility for past failures or successes. Prospective responsibility to tackle a particular issue may be assigned for various reasons. One is the fact that it is a formal part of an official role [13, 14, pp. 212–214, 15]; for instance, perhaps it is constitutive of national government that it has obligations to coordinate a national emergency response. As Cane [13, p. 286] notes, role responsibility is particularly interesting in a political context because a person can be held *liable*, due to their role, for events or the actions of others over which they had no control.

Another important reason is that a particular individual or institution is best placed to address an issue. Thus, one might think that some kinds of personal behaviour are impossible for government to monitor *en masse*, and are thus partly matters of personal responsibility. Finally, prospective responsibility to deal with an issue may come from *causal* contribution to it, subject to conditions of control and knowledge [16]. Thus, government might be responsible for addressing certain outcomes if its own policies contributed to them.

Each of these reasons may be relevant to retrospective responsibility. As we understand it, retrospective responsibility concerns attributions of fault or credit for situations on the basis of an earlier, prospective responsibility. If someone fails to tackle an issue for which they have role, capacity or causal responsibility, they may be retrospectively responsible for this failure.

Our primary interest is in prospective responsibility, particularly exploring how responsibilities should be understood at the outset of the *next* pandemic, should it occur. But this is most usefully done, we think, by considering the question of retrospective responsibility in the current pandemic. We use lessons from the ongoing COVID-19 pandemic to draw conclusions on this issue. The idea of retrospective responsibility is also important because one form of institutional responsibility for which we argue is that institutions must *acknowledge* negative retrospective responsibility rather than attempting to hide it. One important form of institutional responsibility is taking ownership of past failures.

A final caveat is necessary regarding ‘institutional responsibility’. There are important questions about how we should understand the idea that institutions or other corporate bodies, which are made up of a multitude of individuals and the interactions between them, should be understood [17]. We side-step these questions, and in particular the issue of whether institutional responsibility is reducible to the responsibilities of individual members or representatives, or whether we can non-reductively hold institutions themselves responsible. That the former possibility is a live option explains our decision to contrast institutional with ‘personal’, rather than ‘individual’ responsibility.

## Balancing Responsibilities in a Pandemic

A prominent feature of the UK government’s COVID response has been a reliance on slogans, particularly the instruction first adopted in March 2020 [18], and repeated intermittently, to:

Stay at Home. Protect the NHS. Save Lives.

Thus, the instruction for people to avoid contact with one another as far as possible was framed as a personal and collective responsibility for the public. This had two linked goals: protect a highly trusted [19, 20] national institution; and reduce avoidable deaths and serious illness. Other countries’ messaging had a similar emphasis on personal responsibility [21, 22, p. 1, 23], including some of those who appear to have tackled the pandemic more effectively [24]. At least in the UK, the same cannot be said for institutional responsibility. As well as Johnson’s rhetoric noted above, there are many examples of government or other institutions downplaying institutional responsibility, and of failing to meet key institutional responsibilities, some of which we discuss in more detail below [10, 25–29].

This section sets out our view of how various facets of pandemics are subjects of institutional and personal responsibility. A straightforward approach would be to divide up spheres of influence between the personal and the institutional. This section begins this task, but then Section 4 notes two ways in which personal and institutional responsibility are interrelated. In what follows, we consider mistakes made during the COVID pandemic with a view to establishing prospective responsibilities for the future.

### Institutional Pandemic Responsibilities

It is not easy to make the right choices in a novel pandemic. The earliest months of COVID provide a case in point: governments were forced to, as Dutch leader Mark Rutte put it, “mak[e] 100% of the decisions with less than 50% of the information” [30, p. 190]. In any unfamiliar emergency, it would be unreasonable to expect institutions to get everything right [31]. However, this does not preclude three broad types of institutional responsibility. First, while some mistakes are understandable early on, this does not extend to all kinds of error. While no institution can be criticized for failing to perfect their pandemic response immediately, institutions which fail to take lessons from both other countries’ decisions and their own past mistakes are culpable. Institutions thus have a responsibility to properly prepare for pandemics. Second, even if mistakes are inevitable, the outcomes of those errors may be exacerbated by failures to properly respond. Thus, institutions have a responsibility to identify mistakes and change course. Finally, for both culpable and non-culpable mistakes, there is a separate institutional responsibility of honest and effective communication.

The first set of institutional responsibilities relate to pandemic preparedness. Although no pandemic is singularly predictable, what is predictable is the *risk* of serious pandemics [32]. The COVID pandemic was pre-empted by recent outbreaks of Ebola, bird flu and swine flu. Government and health institutions have responsibilities to prepare for such eventualities by having detailed pandemic plans, adaptable to a variety of situations; one criticism of the UK government’s early COVID response was that it was modelled too closely, and for too long, on preparations for an influenza pandemic [33, 34], and involved a general failure to adapt preventive measures quickly enough, leading to a situation where, as Cairney [35, p. 18] puts it “In comparison with many countries, UK government ministers seemed reluctant to enforce state quarantine measures”. Moreover, there is evidence that the UK government *did* engage in pandemic preparedness exercises, but then largely ignored the results [36, p. 503].

Responsibilities of pandemic planning include having appropriate supplies of Personal Protective Equipment (PPE) for healthcare and other frontline workers. They also require government institutions to ensure that key social services, such as the health service and police, are not operating under severe pressures that could turn into disasters in a pandemic. It is worth noting when it comes to these latter two responsibilities that they cover not only the immediate resources required for healthcare professionals and institutions, but also critically reflecting on broader strategies and goals of government. For instance, Sanders [37, p. 368] notes that in the years leading up to COVID, “Emergency stockpiles of PPE had severely dwindled and gone out of date after becoming a low priority in the years of austerity cuts”. Similarly, Oliver [38, p. 1], focusing on care home deaths, suggests that, “Even before the pandemic, [people] had repeatedly highlighted the crisis in care home capacity, funding, financial viability, and inconsistent support from overstretched local NHS services”, noting the responsibility not only of government but of other social institutions such as the press for ignoring this situation (see also [39, p. 33]). This highlights the institutional responsibility to consider whether politically attractive policies such as spending cuts might impact core areas of responsibility.

This links directly to the implication with which we began this section, that it is the responsibility of individual members of the UK public to ‘protect’ the NHS. Whether or not this is accurate in some sense, it obscures the question of why the health service needed so much protection. A public health sector is primarily the responsibility of government, and of sector managers. Thus, as we discuss in more detail in Section 4, it may be accurate to say that the public have a responsibility to ‘protect the NHS’; but this responsibility is situated within broader institutional responsibilities.

Perhaps less obvious—at least, it would not have occurred to us prior to this pandemic—is the question of whether other institutions should also have pandemic preparedness plans. COVID-19 has been one of the most significant upheavals to working life that wealthy countries such as the UK have seen in decades, probably since the Second World War. Many organisations and individuals responded in haphazard ways. For instance, the university sector has been criticized for insisting on a ‘business as usual approach’ [40] and abandoning students [41], though this must be seen in a context of many individuals doing their best in difficult circumstances. One possibility is that many educational institutions are locked into particular models of teaching and learning, perhaps for understandable reasons, which made the transition more difficult than it needed to be. It may be that our new knowledge of the possibility of dramatic and sudden change means there is now a responsibility to engage in ‘future-proofing’ against similar disruptions in years to come.

Pandemic preparedness, by its nature, is an institutional responsibility that occurs before any emergency has begun. We now turn to institutional responsibilities that operate in the ‘moving present’ of an ongoing pandemic. Governmental institutions have various responsibilities to take measures to combat the spread and impact of disease, as well as the effects of those measures. Many of these responsibilities were undertaken by the UK government and its advisors during COVID. State institutions funded research into treatments aimed at alleviating symptoms and reducing deaths [42], and into vaccinations [43], and later took on the complex task of administering those vaccinations. A ‘test and trace’ system was established, and a border control scheme instituted which required quarantine on entering the country, both with the aim of catching cases before others were infected. Government sourced PPE for healthcare and other frontline workers, and developed and disseminated guidance and legal frameworks designed to help direct individual behaviours in ways most beneficial to controlling the spread of the virus. Meanwhile, the Treasury also embarked on a scheme of financial support to meet the severe costs incurred by businesses which were forced to shut during the worst periods of the virus [44]. The government engaged in some financial contributions towards the provision of vaccinations to poorer countries through the COVID-19 Vaccines Global Access (COVAX) initiative [45], though at the time of writing there are still extreme inequalities in levels of vaccination between wealthy and poorer countries. Many of these undertakings required coordination with other governments and international bodies. These are, we suggest, the core institutional responsibilities of government during a pandemic.

Anyone who lived in the UK during 2020 will be struck by two things about the preceding paragraph. First, it is all technically true. Second, if your only information about the country’s pandemic response was that paragraph, you would be poorly informed. While some elements of the government’s response have been praised [31, pp. 11–19], much has come under considerable criticism. To take just a few examples, the government’s advice was repeatedly criticized for being unclear and, at times, self-contradictory [46], while the behaviour of individual people at the top of government was sometimes at odds with the official line, which some research suggested filtered down to reduced public compliance [47], and which came on top of other inconsistencies or confusions in messaging [48, p. 530]. Logistical decisions were criticized for being overly centralized, often ignoring existing local networks [25, 48, pp. 524–525] and relying on targets that served political rather than public health goals [31, p. 4]. The implementation of quarantine for international travelers did occur, but only 12 months after the start of the pandemic, and then only inconsistently [36, p. 504]. Financial support was patchy, with gaps for the newly self-employed and considerable losses to fraud, while there was reluctance to provide meaningful support for those who were supposed to self-isolate, even where for many of those with insecure contracts, unsympathetic bosses, or no sick pay, taking two weeks off work will have been a daunting demand [35].

This brings us to our third category of institutional responsibility. Inevitably, discussing this in the present, practical context highlights the messiness beneath the conceptually neat distinction we made above between prospective and retrospective responsibility. In the middle of an ongoing crisis, a single issue may be the subject of both past choices and forward-looking decisions. These two issues are linked; it is more difficult (though not impossible) to change course on a particular issue if there is no acknowledgement of past error. Attempts to do so may result in public confusion and scepticism. In contrast, highlighting where things have previously gone wrong can provide a clear rationale for changes that can be understandable by those subject to them.

One partial success in this regard is Singapore. In September 2020, Prime Minister Lee Hsien Loong acknowledged that the country had made mistakes in its initial approach, including:

- Not encouraging mask use early enough;
- Not addressing issues with workers living in dormitories; and
- Not quarantining all returners from abroad and not testing them before they left quarantine.

Some of these issues (masks, quarantine) were understandable, and down to international uncertainty about the extent of asymptomatic transmission [49]. But the dormitory issue was predictable—all communal living situations are a risk. Singapore subsequently modified these policies; but Lee has admitted the error, adding that “in the fog of war, it is not possible always to make the perfect decisions” [50]. We do not suggest that Singapore’s COVID response has been perfect since then [51], but that this highlights the way in which governments and other institutions can reasonably acknowledge their failures of responsibility, and publicly identify ways to fix them in the future. In contrast, while the UK government has occasionally, and under pressure, admitted mistakes [52], this has come within the much broader context of deflection.

Regardless of one’s judgement of how particular governments have performed, we suggest that institutions have a responsibility to be up front about what they have got wrong. As Intisar Chowdhury, son of an NHS doctor who warned of PPE shortages before dying of COVID, put it during a radio phone-in involving Hancock:

The public is not expecting the government to handle this perfectly. We just want you to openly acknowledge there have been mistakes in handling this virus. [53]

Chowdhury makes a strong case for pragmatic reasons to acknowledge error: the general public will be aware of errors, inconsistencies, and changes in advice. The (perceived) legitimacy of rules and advice depends on clear admission of previous mistakes, and explanations of how such mistakes are being avoided. There are also moral reasons [54, p. 7]. In a democratic society, citizens have a right to transparent public institutions in all but exceptional cases in order to exercise their democratic rights. Where institutional errors lead to avoidable death and suffering, there is an independent moral requirement based in respect for those who have suffered for institutions to acknowledge their retrospective responsibility for decisions that led to such harms. This is a key area where the UK government has come under criticism [37, p. 356]. While Johnson has promised an inquiry, this commitment was not made until May 2021, and proceedings will not begin until Spring 2022 [55].

As well as having a responsibility to admit what has gone wrong, public-facing institutions also have a responsibility (accepted rather more readily) to acknowledge what has gone right. Again, there are also pragmatic reasons—it is important for people to recognise when progress is made—and moral reasons in that the requirement of honesty cuts both ways. But this more welcome responsibility for institutions to communicate good news suggests a further pragmatic reason to be honest about past errors: when institutions imply they have made no mistakes, it is harder for individual citizens to distinguish genuine successes. Attempts to present an entirely positive story about institutional responsibility may create general cynicism about all institutional claims.

### Personal Responsibility and Its Limits

Just as there are issues during a pandemic for which institutions most properly take responsibility, so too are there issues for which personal responsibility is the more appropriate focus. This section focuses primarily on individuals who do not have any additional special roles during a pandemic; that is, individuals *qua* members of the public. However, Section 4 briefly touches on whether medical professionals have a responsibility to work in situations where they lack adequate protection.

There is some evidence that the UK public have increasingly accepted the role of personal responsibility as the pandemic progresses [47], though this does not mean they do not also hold institutions responsible [56]. However, there is also evidence that most people believe their individual compliance with rules is above average [57, p. 3].

In the absence of extreme levels of coercion, individuals must take responsibility for following reasonable and well-communicated guidance to spreading disease. As Timmerman [54, p. 2] puts it, a pandemic “requires a collective effort that cannot succeed by governmental actions alone and requires the active cooperation of the general population”. In the case of COVID, the evidence suggests that the primary method of infection spread is via air droplets; and so, individuals have a responsibility to avoid sharing unventilated spaces with others where possible, and to wear masks and keep a reasonable distance when this is unavoidable [39, p. 38]. There is also a responsibility to self-isolate if you have evidence that you have contracted the SARS COV-2 virus, and to be honest in dealing with public health officials trying to track the spread of disease.

We also suggest that, if an effective vaccine is available, many individuals have a moral responsibility to get vaccinated (see also [58, pp. 136–137]). The rationale for this is the same as for other preventive measures. For most of us, the risks of vaccination are extremely low. As is the case with COVID, effective vaccines reduce one’s own likelihood of becoming ill, and thus further stretching the resources of health services. The effect on transmission, and thus the risk of harming others, is still a subject of research in the case of COVID, but also seems likely to be significant [59]. Insisting on this moral responsibility does not equate to claiming that government may legally enforce vaccination. While Savulescu [60] argues in favour of mandatory vaccination in some cases, we do not take a position on this issue in this paper.

Even focusing on moral rather than legal responsibility, these claims need qualification. In philosophical discussion, moral responsibility is tied closely to praise and blame; if one fails one’s responsibilities, one is thereby blameworthy (even if it would be counterproductive to actually express blame). Yet it is important to recognize the varying degrees of ease with which people can understand and take on the personal responsibilities we set out above. Recall that on the standard philosophical view responsibility requires both control and knowledge. Knowing about and fulfilling one’s responsibilities may be easy for some, but difficult for others. For instance, the ease with which people can self-isolate upon finding they have symptoms depends on, *inter alia*, their financial stability [23, 61, 62]. The UK government offered limited financial support of £500, though only from September 2020 [63], to a subset of self-isolators, but low-paid individuals who lack benefits such as sick pay will be left significantly vulnerable if they must miss work for an extended period.

Turn now to the knowledge condition on responsibility. According to this condition, you must be capable of reasonably anticipating the effects of your behaviour to be responsible for those effects. As the UK’s COVID vaccination program moves forward with a significant level of success, many have raised worries about the comparatively low level of vaccination among individuals from some ethnic groups. This may be partly attributed to issues that are outside of individual control, but some have suggested that there is also a higher degree of ‘vaccine hesitancy’ in some communities. Vaccine hesitancy does not involve outright opposition to vaccination, but rather may stem from various uncertainties around the speed of development, mistrust of authorities and institutions that may not have served members of one’s ethnic or racial group well, or exposure to misinformation. While individuals have personal responsibilities to consider information critically, Razai et al. [64] note that it is also important to acknowledge the legitimate distrust that some have in medical and other institutions due to personal experiences of discrimination, knowledge about under-representation in medical trials, or of past unethical research (see also [54, p. 8]). It is important to note that these various experiences ground *reasonable* doubts about vaccinations, which may be overcome in many cases through respectful engagement with people’s concerns. If a person’s hesitancy over vaccination is based on such concerns, then in many such cases those individuals will not be responsible for a failure to get vaccinated as soon as possible. But this does not extend to just any refusal to be vaccinated, such as those refusals based on wild conspiracy theories. Such beliefs are *not* reasonable, and so those who believe them still meet the knowledge condition, in that they have no good reason for false beliefs about their personal responsibilities.

We also suggest that individuals have personal responsibilities during a pandemic not only to worry about what is legal, but what is sensible and ethical. Individuals have obligations to evaluate the situation independently and come to their own conclusion about whether a legally sanctioned activity is ethically permissible. This is not always easy; in the context of rapidly changing rules and complex scientific advice, it is understandable that some might fall back on legal permissibility. Yet even the UK government advocated against this at some points. For instance, the government reversed their long-advertised stance that restrictions would be relaxed for Christmas shortly before the celebration, a policy which had come under considerable criticism [26]. This led to scenes at London’s St Pancras station where crowds rushed to get trains to see family while it was still legal to leave the capital [65]. More generally, the government’s policy on new restrictions was largely to announce them several days in advance. Although more could have been done to communicate the difference between legal restrictions and sensible behaviour, it is not beyond the ability of most people to work out that if something is sufficiently risky to warrant legal restrictions in three days’ time, it is probably sufficiently risky to avoid it even before the law changes. Indeed, Newton [36, p. 510] notes, that the UK public was largely “a low trust and sceptical population socially isolating and distancing itself, sometimes before the government told them to and often continuing to do so after the rules were eased”.

A final issue takes us in the opposite direction: is it sometimes permissible to be *less* cautious than advised or legally required? Of course, some people regard the pandemic as a hoax, and thus believe all measures to be unjustified. Since the pandemic is clearly *not* a hoax, much of their behaviour was itself a violation of responsibilities. There are two more interesting types of case. First, some may regard government rules as generally sensible but justifiably believe themselves to be a valid exception to individual rules. For instance, during periods when it was illegal (with some exceptions) to visit others at home, two family groups might have self-isolated carefully for several weeks, and then reasonably believed limited contact to be safe. Second, and relatedly, some people may regard the general *set* of laws as justified but reject particular rules. For instance, there were several periods in the UK’s frequently changing regulations when it was illegal to meet a friend for a walk outside, even though the risks of spreading the virus outside, in small groups, are widely agreed to be low [66].

However, even where the standard reasons to follow the rules are lacking there are still considerations supporting some personal responsibility to follow the law. First, even if a particular individual could be certain they posed no risk of infection, there is no easy way to communicate this to others, and seeing apparent violations by others may weaken people’s motivation to comply themselves. Second, we are often motivated to deliberate in self-serving ways, and to assess risk on the basis of what we would like to be true. Still, there may be cases where following the rules strictly might itself cause considerable harm. Thus, there is no simple account of people’s personal responsibilities around rule-following. While there are some reasons to stick to the rules even when one is reasonably confident that one will not cause harm by failing to do so, the costs of compliance are also relevant.

## The Institutional and the Personal

We have so far suggested that there are some issues that arise in pandemics which can properly be seen as institutional responsibilities, and others which are more appropriately seen as personal responsibilities. This section acknowledges a complexity to this division: personal responsibilities are in many ways shaped *by* the institutional response. Indeed, we argue that institutional responsibility is prior to personal responsibility in a pandemic.

This shaping occurs in two ways. First, the most appropriate way to think about personal responsibilities must presume an adequate institutional response. In other words, many personal pandemic responsibilities apply only if they are situated within a system of effective institutional responsibility. Second, however, the absence of proper institutional responsibility does not automatically obviate personal responsibility. Rather, in some cases of institutional failure there are *remedial* responsibilities which fall on the rest of us as individuals.

### How Institutional Responsibility Structures Personal Responsibility in a Pandemic

Response to a pandemic is a collective action problem. Each individual’s behaviour (e.g., seeing friends in a park) may contribute little to the outcome but collectively such behaviours may have massive costs. While social norms play a part in solving the tragedy of the commons [67], institutional responses are important in structuring those norms and going further to create direct incentives and disincentives. In some cases, laws may be required to restrict behaviour. Such coercive measures are justified when they impose small costs for large collective gains [68, 69].

We noted in Section 3 that it is important when thinking about personal pandemic responsibilities to acknowledge that various responsible behaviours may be difficult to perform for some. Such difficulties do not occur randomly; they are social phenomena structured by institutional responses. The degree to which institutions meet their responsibilities can shape the ability of individuals to meet *their* responsibilities. At the extreme, institutional failures may *undermine* personal responsibilities by affecting either individuals’ ability to carry out relevant actions, or knowledge of what to do.

One clear example of an effect on ability concerns government financial support for self-isolation, mentioned above. Consider what happens if governments fail to provide adequate financial support for individuals who need to self-isolate. In the UK and elsewhere, many businesses closed, and their staff were placed on ‘furlough’. But not all workers were eligible for furlough, e.g., because their workplaces stayed open. Among that group, some were employed on ‘zero hour’ contracts that do not include sick pay, and which require them to accept the shifts they are given or risk losing future employment.

Individuals in this position may disagree with an instruction to come into work when unwell; but for those who have no power to challenge company policy, and no alternative source of income, there may be little choice. As we note above, the UK government only introduced a support payment for those on low incomes six months into the pandemic, and then it was only £500 for the whole period of self-isolation. In one sense, this is a straightforward example of our earlier point: self-isolating is more difficult for those in precarious economic circumstances. But it is also an example of how institutional decisions may impact personal responsibilities. By rendering workers financially unable to self-isolate or avoid interpersonal contact governments, and individual businesses, may change those workers’ responsibilities.

A related effect can be seen in a narrower healthcare setting. In the UK, the NHS has an institutional responsibility to look after those who fall ill during a pandemic. This gives rise to many instances of personal responsibility on the part of individual doctors, nurses, and other healthcare and support professionals. Such personal responsibilities are, arguably, a constitutive part of the professional role such individuals inhabit. Yet these responsibilities are not unconditional. One important difference to the usual caregiving role in a pandemic is the increased personal risk which healthcare and other frontline workers face; for instance, Amnesty International [70] have found that at least 17,000 healthcare workers have died of COVID worldwide, while research has suggested that UK healthcare workers at at much greater risk of ‘severe’ reactions [71]. We have already mentioned issues around the government’s procurement of PPE, which included the purchase of substandard protective equipment. In line with Schüklenk [72], we suggest that healthcare workers’ caregiving responsibilities are conditional on the government and institutional NHS meeting *their* responsibilities to adequately protect workers (see also [39, p. 68]). Like other forms of moral obligation, personal responsibility for behaving in a particular way is subject to a ‘demandingness’ condition [73], where one cannot be expected to fulfil what would otherwise be a responsibility if the costs of doing so are too great.

Additionally, where there are inadequate social measures in place to enable people to comply with personal responsibilities, a rhetorical insistence *on* such personal responsibility is unethical. We have outlined above how the UK government’s advice to the public was criticized for being unclear and confusing, and how some government policies themselves may have worsened the pandemic [29], a view apparently shared by many senior figures in government [74]. In part, this seems to have been due to a lack of clarity within government about who was ultimately responsible for certain decisions [31, p. 28]. Yet this did not stop senior figures and high-profile institutions from pushing the message that it was a failure of *personal* responsibility by the public that was to blame for further surges in infections [8]. Certainly, there will be individuals – perhaps many – who would have ignored even clear advice. But whereas it is reasonable to insist on a combination of institutional and personal responsibility, it is unethical for those at the head of institutions to focus entirely on purported failures of personal responsibility, and to refuse to acknowledge failures of institutional responsibility.

We suggest that a better way for institutions to frame the situation in a pandemic may be to focus on the idea of *shared* responsibility, or a deal between institutions and individuals. It is perhaps natural for those in charge (whether elected or appointed) to want to emphasise successes and minimise failures, and thus easy to focus instead on what the public must do. An alternative framing is the one we have suggested, where institutional responsibilities are clearly set out as providing the conditions to enable people to act in responsible ways at a personal level. In many cases, it is only then that government can reasonably hold individuals accountable.

It is worth briefly explicating how we understand the idea of ‘shared’ responsibility, and in particular how it differs from nearby ideas such as ‘collective’ responsibility, and the concept we have already used of ‘institutional’ responsibility.^1^

Following Smiley [17], we understand the idea of collective responsibility as taking a stance on an issue we explicitly sideline above, namely the question of whether groups or collectives can be understood as moral agents, by locating “the source of moral responsibility in the collective actions taken by these groups understood as *collectives*”. The notion of shared responsibility aims not to take a stance on this issue; thus, ‘the government’, ‘the state’ and ‘the public’ may or may not be irreducible collectives.

Also crucial to our idea of shared responsibility in this context is the view that, although ultimate responsibility for an outcome is shared among relevant parties, there are clearly distinguished roles for each. Responsibility for mitigating pandemics is shared by (individual members of) the public and (individuals in positions of power within) institutions; but as we have described it, there is a clear allocation of responsibilities contributing to that shared goal.

Finally, we can distinguish shared responsibility from ‘institutional’ responsibility by noting that in the case under discussion, one of the major groups involved (the public) is not an institution. While shared responsibility as we have defined it might well operate at both an intra-institutional and inter-institutional level, it ranges more broadly than that because it can also encompass (as it does in the case we focus on) responsibility that is shared between institutional and non-institutional actors, or indeed in situations where no institutions are directly involved.

Turn now to the potential effect of institutional responsibility failures on individuals’ knowledge. A key example of this in the context of the UK’s COVID response is handwashing. Early in the pandemic, the mechanisms by which the SARS CoV-2 virus spread were, understandably, poorly understood. Early public health advice relied heavily on knowledge about the spread of influenza, and so focused on transmission by touching infected surfaces, and thus on hand-washing and other forms of hygiene. We now know that SARS CoV-2 is extremely unlikely to be spread this way, and that the far greater risk is from air droplets. Yet publicly available advice was slow to change and, at the time of writing in June 2021, still places key measures such as ventilation far below less important measures such as handwashing (NHS 2021) [75]. There is some evidence that this has damaged public understanding of responsible behaviour. For instance, a February 2021 poll of 2,331 adults found that while most people saw the virus as primarily transmitted by air, 16% believed that touching infected surfaces was the primary method of transmission [76].^2^ Similarly, Dixon et al. [56], writing in July, found 85% of respondents blaming “failure to keep two metres apart and poor hand-washing” for viral spread.

While it is possible for many individuals to do their own research, and to find out for themselves about the relevance of various preventive measures, doing so takes time, a degree of scientific literacy, and a realization that institutional advice is misleading. Thus, institutional failures of responsibility at least mitigate, and may even fully excuse, failures of a *prima facie* responsibility to take appropriate preventive steps such as ventilating rooms and avoiding indoor meetings.

### Institutional Deficits and Their Implications for Personal Responsibility

The examples considered in Section 4.1 highlight how failures of institutional responsibility may diminish personal responsibility during a pandemic by reducing people’s ability to behave in particular ways, their epistemic access to relevant information, or by increasing the personal costs of fulfilling responsibilities beyond a reasonable threshold. One might think that, given the relationship we have outlined between institutional and personal responsibility, this is the only direction in which this influence occurs.

However, institutional failure does not make a pandemic disappear. People will still be at considerable risk of illness and death. Depending on the extent of institutional failure, people may also be struggling financially or with access to food or medicines. As a result, failures of institutional responsibility may generate additional ‘slack-taking’ responsibilities for some individuals.

The idea that people might have a responsibility to pick up the slack when others fail to meet their own responsibilities is contested [77–79]. Before we come to these arguments, though, a caveat is important: even the most enthusiastic proponent of slack-taking during a pandemic must acknowledge that its scope is limited. Consider what would happen if a national government failed to meet their responsibility in securing sufficient vaccines for their population. It would simply not be possible for most individuals to contribute meaningfully, either alone or in concert, to securing a national vaccination supply. Thus, any slack-taking responsibility must be realistic.

However, there are various areas where it seems more reasonable to expect individuals to take up the slack. One such area concerns decision-making around personal behaviour. We have acknowledged that, for some individuals, poor institutional communication may weaken the responsibility for behaving appropriately by weakening their access to reliable information. For many individual members of the public, though, the government is not the only source of information. Although not as detailed as the advice received by government, there was a considerable level of Covid-related information available to the public. As such, it was possible to form a view—as many did—that the UK government’s legal restrictions, and even advice, were insufficiently cautious at various stages. Thus, we suggest, individual members of the public who were able to do so have a responsibility to form their own view of the risks involved in everyday behaviours, and to exercise a reasonable level of caution *voluntarily*. This is a ‘slack-taking’ responsibility because it requires individuals to engage in a level of (i) risk-assessment and (ii) voluntary self-control that would not be necessary if those in institutional power met their responsibilities to control the spread of disease, and to communicate clearly about what is necessary.

A further instance of slack-taking is more familiar, since it also occurs outside of pandemics. In part due to the economic effects of lockdowns, but also in some cases to rising costs of childcare for parents who normally have children in school, many more families in the UK struggled with putting food on the table during the pandemic than is usual [80]. Food banks represent a failure of institutional responsibility: in a wealthy country such as the UK, the government could avoid people going hungry. Yet this also seems a clear case where, amid failures of responsibility at the formal institutional level, individuals may have to accept additional personal responsibility. While some food banks are run by civil society organisations which can themselves be assimilated into the category of ‘institution’, many others during the COVID-19 era were informal agreements among neighbours or informal community groups to ensure that as many people as possible avoided hunger.

In philosophical discussions of slack-taking, some object to the idea that one person’s responsibilities could be affected by the failure of others to deliver on *their* responsibilities. This discussion typically differs from our case, in that it considers equivalently placed agents. For instance, authors imagine three adults (all of whom can swim) by a pool containing three drowning children. If two refuse to jump in, does the remaining one have a remedial responsibility to save all three? In the cases we are considering, on the other hand, the agents in question are very differently positioned. Nonetheless, we think that at least some of the existing discussion can shed light on our question.

In general, those who object to ‘slack-taking’ do not deny that it would be a morally good thing to do; rather, they deny that it can be described as a genuine obligation. Many who take this line of thought object that where there are shared responsibilities, one’s ‘fair share’ is determined on the assumption that everyone acts as they should. Even when others *fail* to act as they should, one’s fair share does not change [77, p. 90]. Miller [78] takes this line in part due to an understanding of obligations as something which third parties can reasonably enforce.

The concept of responsibility, however, is importantly different than that of obligation. Even accepting Miller’s view of obligations, we suggest that responsibilities are not necessarily subject to enforcement by third parties. Rather, what seems central to responsibilities is that an individual can rightly be *held to account* (or, more strongly, blamed) for non-compliance. As defenders of the idea of ‘slack-taking’ note, even if there is a degree of unfairness in expecting people to do more than would otherwise be reasonable simply because someone else has failed to act responsibly, this unfairness may be outweighed by the needs of those to whom the responsibility was owed in the first place [81]. See also [79, 82–84].

We also suggest that *institutional* failures of responsibility are a special case. Public institutions perform many roles, both licit and illicit. One of these roles is to provide mechanisms for efficiently realizing various obligations that exist somewhat independently of them. As Klein [85, p. 2] puts it, democratic institutions are, in part, “substantive mechanisms for organizing different actors, interests and groups in society”.^3^ While various state and other institutions are the most effective vehicles for the realization of many responsibilities that arise during a pandemic, these responsibilities are ones which we all owe to each another, and which are often best *delivered* through various institutions. If these institutions sometimes fail to deliver, the responsibility remains, and reverts to us (collectively and individually) to solve.

There is one important lesson, however, from objections to slack-taking. We suggested that institutional failures can give rise to new personal responsibilities for which we can be held accountable. But it would not be reasonable for representatives of those very institutions which failed *their* responsibilities to do this account-holding, let alone blaming individuals who in turn failed newly generated personal responsibilities. Rather, moral account holding must come from other similarly placed individuals in the community.

## Conclusion

Responding to a pandemic is primarily an issue of collective responsibility. This requires establishment of norms, and incentives and disincentives including laws, to change human behaviour. It is the responsibility of institutions, particularly governmental institutions, to step up to this task and execute their institutional responsibilities ethically. Perhaps less obviously, it is also essential that they set the conditions—by providing information, ensuring understanding, and providing tangible opportunity—for individuals to meet their personal obligations, both legal and ethical. Although some focus almost exclusively on institutional responsibility, personal responsibility does exist in a pandemic, and under some circumstances its scope extends beyond responsibilities set by government and other institutions. But, given the collective nature of a successful pandemic response, institutional responsibility is prior to personal responsibility. Governments need to take responsibility, admit failures of responsibility openly, and shift course as mistakes become clear. They should not engage in “victim blaming” but enable their citizens to be responsible agents.

## Footnotes

1 We thank an anonymous reviewer for pushing us to clarify here.

2 Ritchie does not link directly to the polling data he cites, nor is it publicly available online, but we have independently verified it with the polling group responsible, Comres-Savanta.

3 Though as Klein [85, p. 26] notes, we should not make the mistake of seeing the state as “a neutral medium for implementing collective decisions”, since it is also a political entity with its own set of interests.
